# Reduced Endothelium-Dependent Relaxation to Anandamide in Mesenteric Arteries from Young Obese Zucker Rats

**DOI:** 10.1371/journal.pone.0063449

**Published:** 2013-05-07

**Authors:** Nubia S. Lobato, Fernando P. Filgueira, Roshini Prakash, Fernanda R. Giachini, Adviye Ergul, Maria Helena C. Carvalho, R. Clinton Webb, Rita C. Tostes, Zuleica B. Fortes

**Affiliations:** 1 Department of Biological Sciences, Federal University of Goias, Jatai, GO, Brazil; 2 Department of Physiology, Georgia Health Sciences University, Augusta, Georgia, United States of America; 3 Department of Pharmacology, Institute of Biomedical Sciences, University of Sao Paulo, Sao Paulo, Brazil; 4 Department of Pharmacology, School of Medicine of Ribeirao Preto, University of Sao Paulo, Ribeirao Preto, SP, Brazil; Idaho State University, United States of America

## Abstract

Impaired vascular function, manifested by an altered ability of the endothelium to release endothelium-derived relaxing factors and endothelium-derived contracting factors, is consistently reported in obesity. Considering that the endothelium plays a major role in the relaxant response to the cannabinoid agonist anandamide, the present study tested the hypothesis that vascular relaxation to anandamide is decreased in obese rats. Mechanisms contributing to decreased anandamide-induced vasodilation were determined. Resistance mesenteric arteries from young obese Zucker rats (OZRs) and their lean counterparts (LZRs) were used. Vascular reactivity was evaluated in a myograph for isometric tension recording. Protein expression and localization were analyzed by Western blotting and immunofluorescence, respectively. Vasorelaxation to anandamide, acetylcholine, and sodium nitroprusside, as well as to CB_1_, CB_2_, and TRPV1 agonists was decreased in endothelium-intact mesenteric arteries from OZRs. Incubation with an AMP-dependent protein kinase (AMPK) activator or a fatty acid amide hydrolase inhibitor restored anandamide-induced vascular relaxation in OZRs. CB_1_ and CB_2_ receptors protein expression was decreased in arteries from OZRs. Incubation of mesenteric arteries with anandamide evoked endothelial nitric oxide synthase (eNOS), AMPK and acetyl CoA carboxylase phosphorylation in LZRs, whereas it decreased phosphorylation of these proteins in OZRs. In conclusion, obesity decreases anandamide-induced relaxation in resistance arteries. Decreased cannabinoid receptors expression, increased anandamide degradation, decreased AMPK/eNOS activity as well as impairment of the response mediated by TRPV1 activation seem to contribute to reduce responses to cannabinoid agonists in obesity.

## Introduction

Obesity is a major public health problem worldwide [1], [2]. This condition is considered one of the main risk factors for the increased morbidity and mortality from hypertension, dyslipidemia, type 2 diabetes, heart failure, stroke, and coronary artery disease [3]. Impaired vascular function manifested by an altered ability of the endothelium to release endothelium-derived relaxing factors and endothelium-derived contracting factors is consistently demonstrated in obese individuals [4], and is considered the first step in the progression of cardiovascular diseases [5]–[7].

Increased circulating levels of endocannabinoid agonists have been reported in obesity. High levels of the endogenous cannabinoids anandamide (arachidonylethanolamide) and 2-arachidonoylglycerol in the plasma of obese subjects is correlated with visceral adiposity [8]–[10]. Endocannabinoids are lipid mediators generated by almost all cell types both in the brain and peripheral tissues. The endocannabinoid system comprises the endocannabinoids, the enzymes involved in their biosynthesis and degradation and the G protein-coupled receptors (CB_1_ and CB_2_) that mediate their effects [11]–[14]. This system plays an important role in the central and peripheral regulation of energy homeostasis, lipid metabolism, and fat accumulation [15].

Studies in normal rodents have identified that the endogenous cannabinoid ligand anandamide lowers blood pressure and heart rate in anaesthetized animals [16], [17]. This compound is also considered a potent vasodilator in a number of isolated vascular preparations, including mesenteric arteries, renal arteries, coronary arteries and aorta [18]–[22]. Some studies have implicated the endothelium in the relaxant response to anandamide, with the release of different endothelium-derived relaxant factors, including prostanoids, endothelium-derived hyperpolarizing factor (EDHF) and nitric oxide (NO) [18]–[24]. Nevertheless, the relevance of the vascular actions of the endocannabinoid system in obesity is still unknown. In the present study, we investigated the implications of obesity for the response to anandamide in resistance mesenteric arteries. Considering that obesity is often associated with vascular dysfunction, we hypothesized that vascular relaxation to the cannabinoid agonist anandamide is decreased in obese Zucker rats. Whether reduced anandamide-induced vasodilation is due to reductions in cannabinoid receptors expression or decreased activation of signaling pathways were also determined. To test our hypothesis, we used resistance mesenteric arteries from 6–7 weeks-old obese Zucker rats (OZRs) and their lean counterparts (LZRs). At this age, OZRs are normotensive and normoglycemic, allowing that vascular responses be determined without the interference of hypertension or diabetes. Various pharmacological tools were used to investigate the involvement of cannabinoid receptors, anandamide degradation as well as the contribution of the endothelial nitric oxide synthase (eNOS) pathway to the reduced responses to anandamide in obesity.

Our results showed that relaxation induced by the cannabinoid agonist anandamide is decreased in endothelium-intact arteries from young OZRs. Reduced cannabinoid receptors expression, decreased anandamide-induced activation of AMPK and eNOS, increased degradation of anandamide as well as impairment of the response mediated by TRPV1 activation might be involved in the decreased response to anandamide in OZRs.

## Methods

### Animals

All animal procedures were performed in accordance with the Guide for the Care and Use of Laboratory Animals published by the US National Institutes of Health (NIH Publication No. 85–23, revised 1996) and approved by the Institutional Animal Care and Use Committees at the Georgia Health Sciences University. Male, six to seven weeks-old lean Zucker rats (LZRs) and OZRs were purchased from Harlan Laboratories and were maintained on a 12-hour light/dark cycle under controlled temperature (22±1°C) with access to food and water *ad libitum*. On the day of the experiment, after food deprivation (for 5 h), LZRs and OZRs were weighted, and tail blood samples were taken. Blood glucose levels were analyzed using a glucometer (Roche, Mannheim, Germany). Animals were killed by carbon dioxide exposure, followed by diaphragm incision and the white adipose tissue (epididymal and retroperitoneal) was dissected and weighed. Blood pressure (BP) was measured in conscious rats by an indirect tail-cuff method (Kent Scientific Corporation, CT) after a training period of three days. Rats were maintained at 37°C for 10 min, and then three consecutive stable BP measurements were averaged.

### Vascular Function Studies

Force development in response to a specific experimental protocol was evaluated in mesenteric arteries from both groups as previously described [25]. The mesenteric vascular bed was removed and placed in modified Krebs-Henseleit solution of the following composition (in mM): 130 NaCl, 14.9 NaHCO_3_, 4.7 KCl, 1.18 KH_2_PO_4_, 1.17 MgSO_4_·7H_2_O, 5.5 glucose, 1.56 CaCl_2_·2H_2_O, and 0.026 EDTA. Segments (2 mm in length) of the mesenteric arteries were mounted on 40-µm wires in a small vessel myograph for isometric tension recording. The vessels were allowed to equilibrate for about 30 min in modified Krebs-Henseleit solution, which was gassed with 5% CO_2_ in O_2_ to maintain a pH of 7.4. The relationship between resting wall tension and internal circumference was determined, and the internal circumference, L100, corresponding to a transmural pressure of 100 mmHg for a relaxed vessel *in situ*, was calculated. The vessels were set to the internal circumference L1, given by L1 = 0.9×L100. The effective internal lumen diameter was determined as L1 = L1/π, and was between 200 and 300 µm. After stabilization, arterial integrity was assessed by stimulation of vessels with 120 mM KCl. Endothelial function was assessed by testing the relaxant effect of acetylcholine (ACh, 1 µM) on vessels precontracted with phenylephrine (1 µM). Mesenteric arteries exhibiting a vasodilator response to ACh greater than 90% were considered endothelium-intact vessels. The failure of ACh to elicit relaxation of mesenteric arteries (which were previously subjected to rubbing of the intimal surface with a human hair) was taken as proof of endothelium removal.

### Experimental Protocols

Cumulative concentration–response curves to anandamide, ACEA (a CB_1_ receptor-selective agonist) JWH-015 (a CB_2_ receptor-selective agonist) and capsaicin (a vanilloid receptor agonist) were performed in U46619-precontracted mesenteric arteries. To determine whether anandamide-induced relaxation was dependent on the endothelium, responses were also determined in endothelium-denuded arteries. In order to investigate if the decreased relaxation to cannabinoid agonists was associated with an overall impairment in vascular function, rather than a specific endocannabinoid response, cumulative concentration–response curves to ACh and sodium nitroprusside (SNP) were performed in U46619-precontracted mesenteric arteries. Each preparation was tested with a single agent.

To investigate the involvement of cannabinoid receptors in anandamide responses, mesenteric arteries were preincubated for 30 min with either the CB_1_ receptor antagonist (AM251, 1 µM) [26] or the CB_2_ receptor antagonist (AM630, 1 µM) [27].

To determine whether reduced responses to anandamide were associated with abnormal enzymatic hydrolysis of the cannabinoid agonist and/or changes in the AMP-dependent protein kinase (AMPK) pathway, anandamide responses were determined in the presence of the fatty acid amide hydrolase (FAAH) inhibitor URB597 (100 nM) [28] or the AMPK activator aminoimidazole carboxamide ribonucleotide (AICAR, 1 mM) [29], respectively. To examine the contribution of NO in the vascular effects of anandamide, mesenteric arteries were pretreated with the NOS inhibitor Nω-nitro-L-arginine methyl ester (L-NAME, 100 µM, for 30 min) [30].

To investigate the involvement of sensory C-fibres in anandamide responses, mesenteric arteries were pretreated with different blockers. Desensitization of C-fibres in vitro was induced using the selective neurotoxin capsaicin (1 µM for 20 minutes, followed by a 40-minute washout period) [31]. Acute exposure to capsaicin promotes activation of sensory C-fibres; however, after prolonged exposure, as in the current protocol, a desensitization of the nerve ending occurs. A 40-minute washout period ensures removal of any residual neuropeptide that may have been released, as previously described [32]. Considering that transient receptor potential vanilloid-1 (TRPV-1) channels have been identified as the major activation site on C-fibre nerve endings [33], we investigated the effects of the selective TRPV1 blocker capsazepine (3 µM, 30 min) [34] and the nonselective cation channel blocker ruthenium red (30 µM, 30 min) [35] on anandamide responses. In addition, the participation of the products released after activation of vanilloid receptors was investigated using the calcitonin gene-related peptide (CGRP) receptor antagonist α-CGRP (8–37) (10 µM, 30 min) [36] or the P2Y1 receptor antagonist MRS2179 (1 µM, 30 min) [37].

Considering that we did not observe any differences in the responses to anandamide between endothelium-intact and endothelium-denuded arteries from LZRs or OZRs, the studies were carried out on endothelium-intact arteries.

### Western Blotting

To test whether anandamide alters the activity of eNOS, AMPK and acetyl CoA carboxylase (ACC) in mesenteric arteries from LZRs and OZRs, vessels from both groups were isolated, cleaned of fat, dissected and incubated with 1 µM anandamide or vehicle for 30 min, and the activation these proteins was examined. In order to investigate the role of AMPK activation on eNOS phosphorylation, mesenteric arteries from LZRs and OZRs were incubated with Compound C (1 µM), an AMPK inhibitor, 30 minutes before the incubation with anandamide.

After the incubation protocols, vessels were frozen in liquid nitrogen and proteins were extracted (50 µg) and separated by electrophoresis on 8% polyacrylamide gels and transferred to nitrocellulose membranes. Nonspecific binding sites were blocked with 5% skim milk in Tris-buffered saline solution with Tween (0.1%) for 1 hour at 24°C. Membranes were incubated with antibodies (at the indicated dilutions) overnight at 4°C. Antibodies were as follows: anti-CB_1_ (1∶250, Sigma), anti-CB_2_ (1∶1000, Sigma), anti–TRPV1 (1∶2000, Sigma), anti-eNOs (1∶500, Cell Signaling), anti-phospho eNOs (1∶1000, Cell Signaling), anti-AMPK (1∶1000, Cell Signaling), anti-phospho AMPK (1∶1000, Cell Signaling), anti-ACC (1∶1000, Cell Signaling), anti-phospho ACC (1∶1000, Cell Signaling) and anti-β-actin (1∶20000, Sigma). After incubation with secondary antibodies, signals were revealed by chemiluminescence, visualized by autoradiography and quantified densitometrically. Results were normalized to β-actin expression and expressed as units relative to the control.

### Immunofluorescence and Confocal Microscopy

After fixation of the endothelium-intact mesenteric artery segments, samples were frozen in OCT compound (Sakura Finetek USA, Torrance, CA), and serial cryosections (20 µm) were prepared and mounted on slides. After washing in phosphate-buffered saline (PBS), slides containing samples were blocked with bovine serum albumin (BSA, 0.1%) for 1 hour at room temperature. The sections were subsequently incubated (at the indicated dilutions) with rabbit monoclonal anti-CB_1_ (1∶100, Abcam), rabbit polyclonal anti-CB_2_ (1∶100, Abcam), or mouse anti-von Willebrand (1∶500, Abcam) overnight at 4°C. After washing in PBS, the fluorescent secondary antibodies (at a 1∶1000 dilution) goat anti-mouse IgG Alexa Fluor 488 and goat anti-rabbit Alexa Fluor 594 (Molecular Probes) were applied and incubated for 1 h at room temperature. After washing in PBS, the slides were coverslipped with anti-fading mounting medium (Gel/Mount medium; Biomeda, Foster City, CA). Fluorescent photomicrographs were obtained using a laser scanning confocal microscope (Leica TCS-DMRE, Germany). The nuclear stain 4′,6-diamidino-2-phenylindole (DAPI) was used to label all cells. No significant fluorescence was observed when the primary antibodies were omitted.

### Data Analysis and Statistical Procedures

Vasodilatation is represented as a percentage of the maximal response to U46619. The individual relaxation curves were fitted into a curve by non-linear regression analysis. pEC50 (defined as the negative logarithm of the EC50 values) and maximal response were compared by t-tests or ANOVA, when appropriated. The Prism software, version 5.0 (GraphPad Software Inc., San Diego, CA, USA) was used to perform the analysis of these parameters as well as to fit the sigmoidal curves. Data are presented as mean ± SEM. N represents the number of animals used. P values less than 0.05 were considered significant.

### Drugs

Phenylephrine, acetylcholine, sodium nitroprusside, AICAR, L-NAME and compound C were purchased from Sigma Chemical Co (St. Louis, MO). Anandamide, ACEA, JWH-015, AM251, AM630 and MRS2179 were purchased from Tocris (Ellisville, MO). URB597 was obtained from Cayman Chemical (Ann Arbor, MI). α-CGRP (8–37) was purchased from Bachem (Torrance, CA).

## Results

### General Characteristics of OZRs

Table 1 summarizes metabolic parameters in young LZRs and OZRs. Although BP and glucose levels were similar between LZRs and OZRs, OZRs displayed higher body weight and fat mass compared with age-matched LZRs.

**Table 1:** Metabolic parameters in young LZRs and OZRs.

Parameter	Lean Zucker	Fatty Zucker
Body weight (g)	193.0±11.1	283.3±18.2*
Retroperitoneal fat mass, (g/100 g)	0.46±0.03	2.05±0.30*
Perigonadal fat mass, (g/100 g)	0.54±0.02	2.3±0.45*
Glycemia, (mg/dL)	122.0±0.8	124.0±2.5
Blood pressure, (mmHg)	110.5±3.6	116.8±1.6
Data are mean ± SEM; *P<0.05 vs. lean Zucker; n = 8/group.		

### Effect of Obesity on Vascular Relaxation to Cannabinoid Agonists

**Figure 1:**
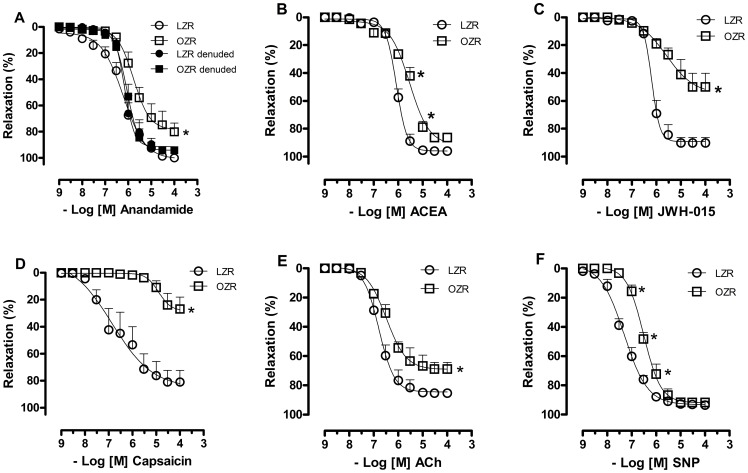
Effect of obesity on relaxation of mesenteric arteries to different agonists. Cumulative concentration-response curves to anandamide (**A**), ACEA, CB_1_ receptor-selective agonist (**B**), JWH-015, CB_2_ receptor-selective agonist (**C**), capsaicin, vanilloid receptor agonist (**D**), acetylcholine (ACh), endothelium-dependent vasodilator (**E**) and sodium nitroprusside (SNP), endothelium-independent vasodilator (**F**) in U46619-precontracted mesenteric arteries from lean Zucker rats (LZRs) and obese Zucker rats (OZRs). Each point represents the mean ± SEM. *, P<0.05 vs. LZR. N = 6/group.

Anandamide induced concentration-dependent relaxation in endothelium-intact mesenteric arteries from both LZRs and OZRs. However, this response was decreased in arteries from OZRs (Emax in %, LZR = 100.9±3.8; OZR = 78.5±4.8, p<0.05; Figure 1A). In endothelium-denuded preparations, relaxation responses to anandamide were similar in arteries from OZRs and LZRs (Emax in %, LZR = 88.4±3.9; OZR = 94.3±2.3, Figure 1A). Endothelium-intact arteries from OZRs also displayed a marked decrease in the vascular relaxation mediated by CB_1_ (pEC50, LZR = 6.1±0.03; OZR = 5.5±0.06, p<0.05) and CB_2_ (Emax in %, LZR = 89.3±2.1; OZR = 54.2±6.9, p<0.05) cannabinoid receptor agonists compared with vessels from LZRs (Figures 1B and 1C). Similarly, the capsaicin-induced relaxation was markedly decreased in OZRs compared with LZRs (Emax in %, LZR = 85.4±12.2; OZR = 28.3±5.1, p<0.05; Figure 1D). A decreased response to the endothelium-dependent vasodilator ACh (Emax in %, LZR = 85.4±1.1; OZR = 69.2±1.4, p<0.05, Figure 1E) as well as to SNP (an endothelium-independent vasodilator) was observed in vessels from OZRs (Emax in % = 69.2±1.4, Figure 1F) vs. LZR arteries (Emax in % = 85.4±1.1).

**Figure 2:**
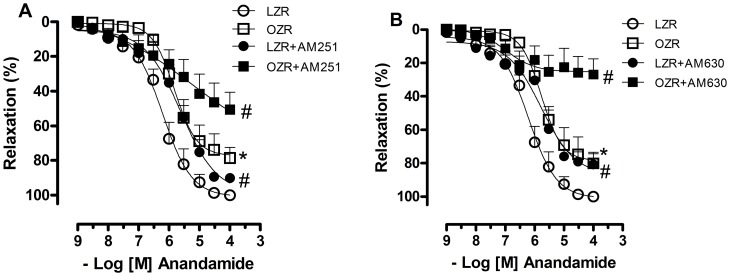
Contribution of cannabinoid receptors activation to the vascular effects of anandamide. In **A**, the vessels were pretreated with a CB_1_ antagonist (AM 251, 1 µM) and in **B** with a CB_2_ antagonist (AM 630, 1 µM) for 30 minutes. The relaxation to anandamide was evaluated in U46619-precontracted mesenteric arteries from lean Zucker rats (LZRs) and obese Zucker rats (OZRs). Each point represents the mean ± SEM. *, P<0.05 vs. LZR. #, P<0.05 vs. respective group in the absence of blockade. N = 6/group.

Blockade of CB_1_ and CB_2_ receptors with AM251 and AM630, respectively, slightly decreased the relaxation responses to anandamide in arteries from LZRs (pEC50, LZR = 6.2±0.09; LZR+AM251 = 5.6±0.1; LZR+AM630 = 5.3±0.1, p<0.05). On the other hand, anandamide responses were considerably reduced in arteries from OZRs after incubation with CB_1_ and CB_2_ antagonists (Emax in %, OZR = 77.3±4.3; OZR+AM251 = 52.9±8.8; OZR+AM630 = 25.3±4.2, p<0.05, Figures 2A and B).

**Figure 3:**
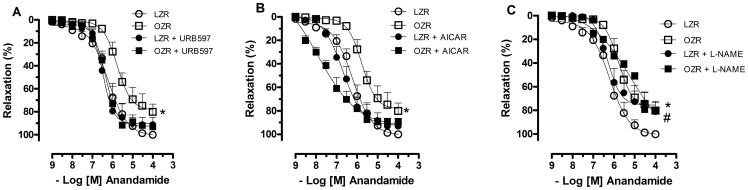
Contribution of anandamide degradation, AMP-dependent protein kinase (AMPK) pathway and nitric oxide to anandamide responses. In A, the vessels were pretreated with the fatty acid amide hydrolase (FAAH) inhibitor (URB597, 1 µM, 30 minutes). In B, vessels were incubated with AICAR (an AMPK activator, 1 mM, 30 minutes). In C, vessels were pretreated with an NO synthase inhibitor (L-NAME, 100 µM, 30 minutes). The relaxation to anandamide was evaluated in U46619-precontracted mesenteric arteries from lean Zucker rats (LZRs) and obese Zucker rats (OZRs). Each point represents the mean ± SEM. *, P<0.05 vs. LZR. #, P<0.05 vs. respective group in the absence of blockade. N = 6/group.

Incubation of mesenteric arteries from OZRs with either the FAAH inhibitor (URB597) or the AMPK activator (AICAR) corrected the reduced relaxation response to anandamide (Emax in %, OZR = 76.04±5.4; OZR+URB597 = 92.6±2.8; OZR+AICAR = 93.4±5.2, p<0.05, Figures 3A and B). Inhibition of NOS with L-NAME significantly decreased the anandamide-induced relaxation of vessels from LZRs, whereas it did not alter this response in samples from OZRs (Figure 3C).

**Figure 4:**
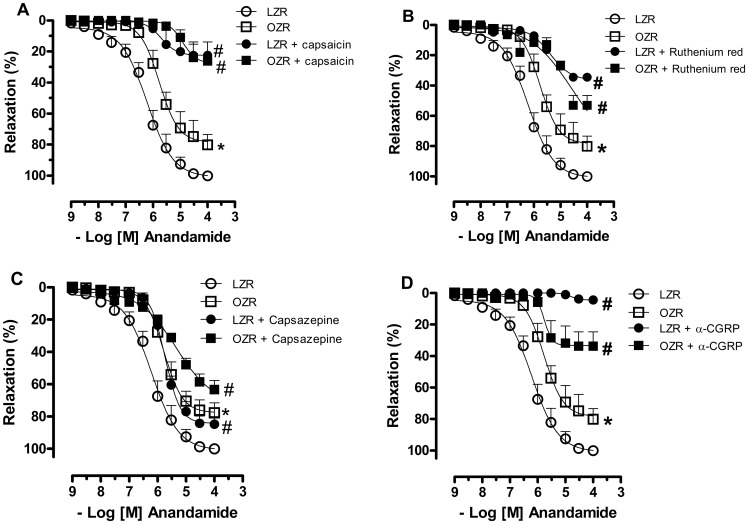
Involvement of TRPV1 receptors on sensory C-fibres in anandamide responses. In **A**, in vitro C-fiber desensitization was induced using the selective neurotoxin capsaicin (1 µM). In **B** and **C**, vessels were pretreated with the TRPV1 blockers, ruthenium red (30 µM, 30 minutes) and capsazepine, respectively (3 µM, 30 minutes). In **D**, vessels were pretreated with the calcitonin gene-related peptide (CGRP) receptor antagonist α-CGRP (8–37) (10 µM, 30 minutes). The relaxation to anandamide was evaluated in U46619-precontracted mesenteric arteries from lean Zucker rats (LZRs) and obese Zucker rats (OZRs). Each point represents the mean ± SEM. *P<0.05 vs. LZR, #P<0.05 vs. respective group in the absence of blockade. N = 6/group.

In vitro C-fiber desensitization with capsaicin markedly reduced the anandamide-induced relaxation of arteries from both LZRs (Emax in %, LZR = 101.0±3.9; LZR+capsaicin = 22.6±3.9, p<0.05, figure 4A), and OZRs (Emax in %, OZR = 77.3±4.3; OZR+capsaicin = 27.7±5.6, p<0.05, figure 4A). Blockade of TRPV1 with either ruthenium red or capsazepine also decreased anandamide-induced vessel relaxation in samples from both LZRs (Emax in %, LZR = 101.0±3.9; LZR+ruthenium red = 42.3±1.8; LZR+capsazepine = 82.7±2.5, p<0.05, figures 4B and C) and OZRs (Emax in %, OZR = 77.3±4.3; OZR+ruthenium red = 46.5±5.6; OZR+capsazepine = 54.7±5.0, p<0.05, figures 4B and C). Purinergic receptors antagonism with MRS2179 did not change anandamide-induced arterial relaxation in vessels from both experimental groups (data not shown). However, the blockade of CGRP receptors with α-CGRP (8–37) almost completely inhibited the anandamide-induced relaxation in LZRs (Emax in %, LZR = 100.8±4.1; LZR+α-CGRP (8–37) = 4.6±0.3, p<0.05, figure 4D), and it decreased this response to a lesser extent in OZRs (Emax in %, OZR = 78.5±4.7; OZR+α-CGRP (8–37) = 33.3±3.2, p<0.05, figure 4D).

### Effect of Obesity on Protein Expression of Cannabinoid Receptors and TRPV1 Receptor

**Figure 5:**
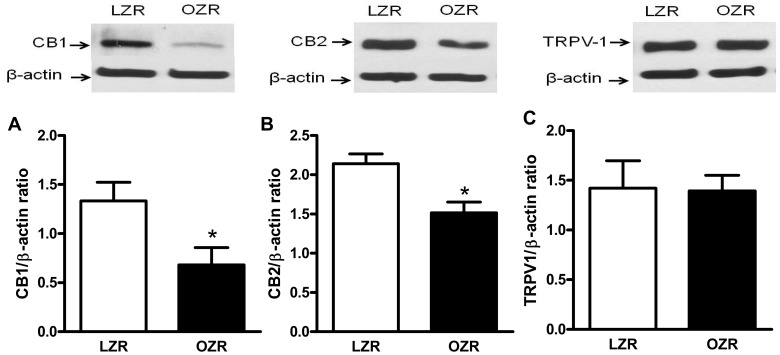
Effect of obesity on cannabinoid receptors protein expression in mesenteric arteries. Panels show densitometric analysis of the Western blots for CB_1_ and CB_2_ protein expression in vessels from lean Zucker rats (LZRs) and obese Zucker rats (OZRs). In **A** and **B**, Western blots for CB_1_ and CB_2_ receptors, respectively. In **C**, Western blots for TRPV1 receptors. Results were normalized to β-actin expression and expressed as units of change from the control. Data are expressed as mean ± SEM. *, P<0.05 vs. LZR. N = 5/group.

CB_1_ and CB_2_ receptors protein expression was decreased in mesenteric arteries from OZRs as compared with LZRs (Figures 5A and B). The protein expression of TRPV1 receptors was similar in mesenteric arteries from LZRs and OZRs (Figure 5C).

### Localization of CB_1_ and CB_2_ Receptors in Mesenteric Arteries

**Figure 6:**
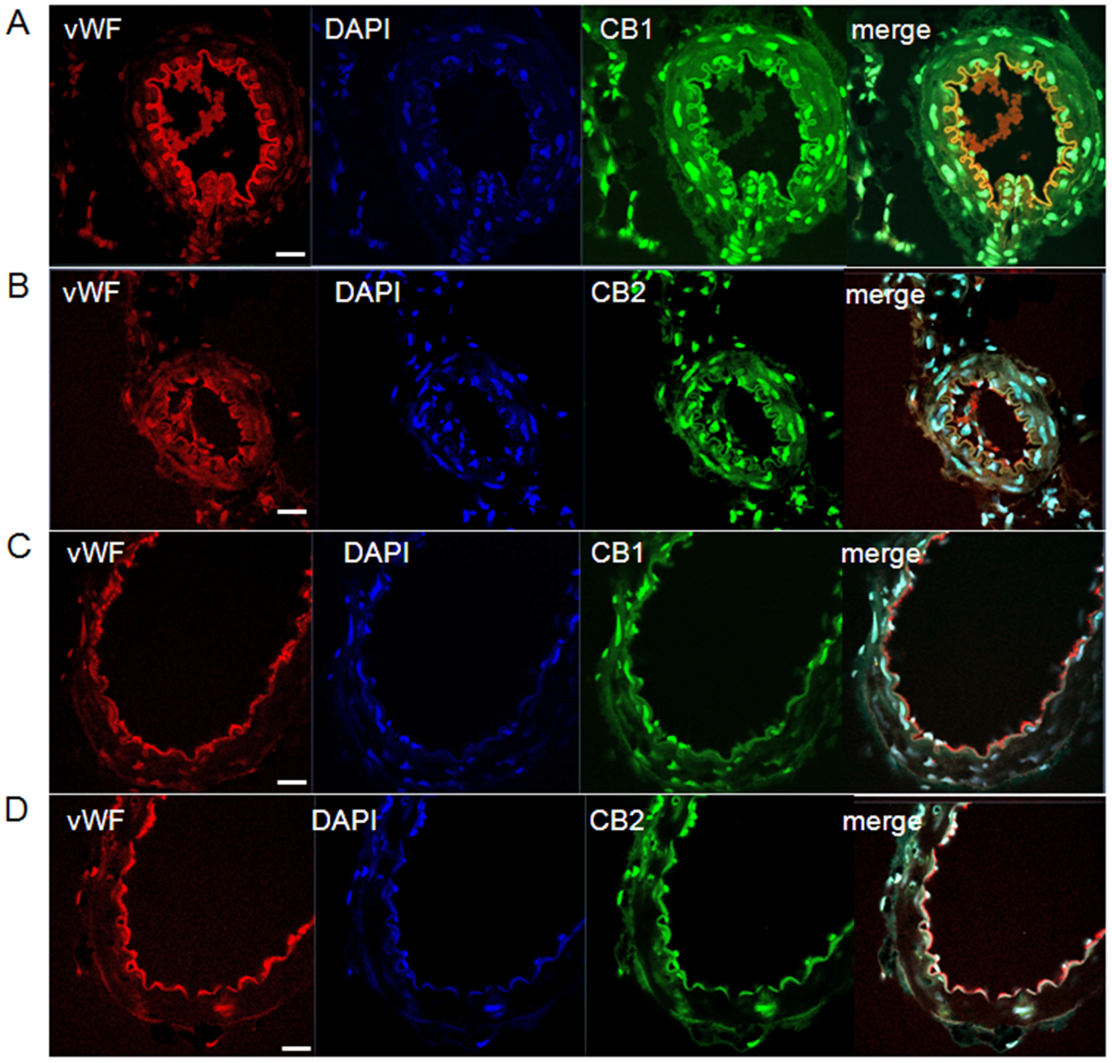
Localization of CB_1_ and CB_2_ receptors in mesenteric arteries. Panels show confocal microscopic images of von Willebrand factor (vWF, red), DAPI (blue), CB_1_ and CB_2_ receptors (green) in mesenteric arteries from lean Zucker rats (LZRs, **A** and **B**) and obese Zucker rats (OZRs, **C** and **D**). Endothelium-intact mesenteric sections were immunolabeled with antibodies against CB_1_, CB_2_ and von Willebrand factor. The nuclei were counterstained with DAPI. An overlay of the vWF, DAPI and CB_1_ or CB_2_ images is presented (merge). Endothelial localization of CB_1_ and CB_2_ receptors is shown in the merged images (yellow). The images are representative of three separated experiments. N = 5/group. Scale bar = 40 µm.

Localization of cannabinoid receptors was determined by confocal immunofluorescence microscopy in endothelium-intact mesenteric sections. Immunofluorescence for CB_1_ (Figure 6A) and CB_2_ (Figure 6B) receptors was visualized in mesenteric arteries of LZRs. To determine whether CB_1_ and CB_2_ receptors were localized in endothelial cells, we used double immunolabeling of rat mesenteric arteries for CB_1_, CB_2_ and von Willebrand factor, a widely used marker for endothelial cells. As shown in Figure 6, CB_1_ and CB_2_ receptors (green fluorescence) co-localized with Von Willebrand factor (red fluorescence), as indicated by the yellow colour of the overlay of CB_1_ or CB_2_ and von Willebrand stainings. Mesenteric arteries from OZRs displayed decreased CB1 (Figure 6C) and CB2 (Figure 6D) immunofluorescence in both smooth muscle and endothelial cells when compared with the representative images from LZRs.

### Effect of Anandamide on the Phosphorylation of eNOS and AMPK in Arteries from LZRs and OZRs

**Figure 7:**
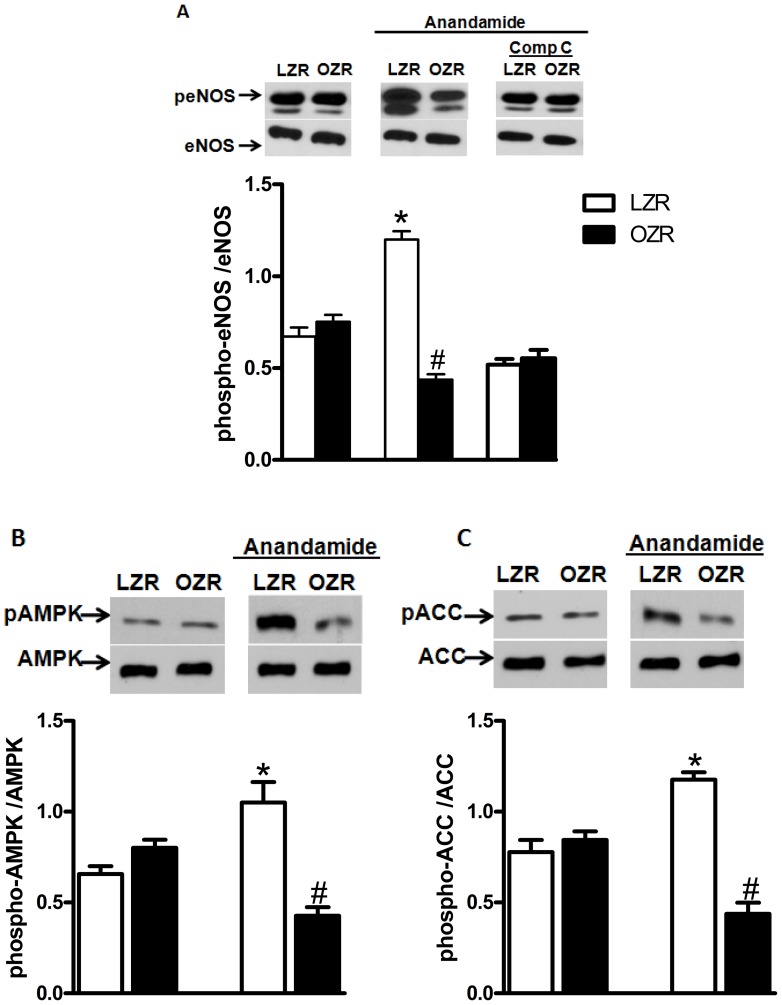
Effect of anandamide on eNOS, AMP-dependent protein kinase (AMPK) and acetyl-CoA carboxylase (ACC) phosphorylation. Panels show densitometric analysis of the Western blots for eNOS AMPK and ACC protein expression in mesenteric arteries from lean Zucker rats (LZRs) and obese Zucker rats (OZRs). In **A**, mesenteric arteries were incubated with anandamide (1 µM, 30 minutes) and the phosphorylation of eNOS at Ser-1177 was examined. Some vessels were incubated with Compound C (Comp C, 1 µM), 30 minutes before the incubation with anandamide. In **B** and **C**, vessels were incubated with anandamide (1 µM, 30 minutes) and the phosphorylation of AMPK Thr-172 and ACC were examined, respectively. Total protein levels are shown as loading controls. Data are expressed as mean ± SEM. *, P<0.05 vs. LZR, #, P<0.05 vs. OZR. N = 5 or 6/group.

Basal phosphorylation of eNOS at Ser-1177 was similar in vessels from OZRs and LZRs. However, after incubation with anandamide, eNOS phosphorylation was decreased in mesenteric arteries from OZRs. In contrast, a robust increase in eNOS phosphorylation was observed in anandamide-stimulated vessels from LZRs (Figure 7A). Incubation of mesenteric arteries from LZRs with Compound C (1 µM), an AMPK inhibitor, 30 minutes before the incubation with anandamide, abolished anandamide-induced eNOS phosphorylation (Figure 7A). eNOS phosphorylation in OZRs was not affected by this inhibitor.

Similarly to the observed with eNOS, anandamide treatment decreased AMPK Thr-172 phosphorylation in arteries from OZRs and increased AMPK phosphorylation in vessels from LZRs (Figure 7B). To confirm that AMPK activation by anandamide results in typical AMPK-mediated downstream responses, we determined the phosphorylation of ACC, a primary target of activated AMPK. We found that anandamide treatment increased ACC phosphorylation in arteries from LZRs. In contrast, phosphorylation of ACC was decreased in vessels from OZRs after incubation with anandamide (Figure 7C).

## Discussion

In the present study we demonstrated that the vascular response to anandamide, an endocannabinoid agonist, is decreased in obesity. Herein, we also present the novel findings that endothelium-intact resistance mesenteric arteries from young OZRs exhibit reduced cannabinoid receptors expression, increased degradation of anandamide, decreased anandamide-induced activation of AMPK and eNOS as well as impairment of the response mediated by TRPV1 activation.

Central nervous system and peripheral abnormalities are observed in obesity contributing to the increased risk for the development of cardiovascular diseases. Of great interest is the impaired vascular function. There is good evidence in isolated vascular preparations that cannabinoids elicit vasodilator effects not only via cannabinoid receptors but also via vanilloid receptors [38], [39]. However, the importance of these effects for the regulation of the vascular resistance and whether these effects are impaired in obesity have not been established so far. Most of the known cardiovascular effects of the cannabinoid compounds are dependent on the underlying conditions. These effects were implicated in the mechanisms underlying hypotension associated to hemorrhagic shock, both cardiogenic and septic, advanced liver cirrhosis, cirrhotic cardiomyopathy, heart failure induced by doxorubicin and shock associated to necrotizing pancreatitis [40]–[44]. Recent evidence also indicates that the endocannabinoid system plays an important role in cardiovascular regulation in hypertension, limiting the elevation of blood pressure and cardiac contractile responses through tonic activation of CB1 receptors [45]. In this regard, it was demonstrated that in vivo treatment with the CB1 receptor antagonist induced a significant increase in cardiac contractility and blood pressure in hypertensive rats. On the other hand, it contributed to decrease blood pressure in weight-loss clinical trials especially in obese patients with hypertension [46]. Altogether these findings suggest that the overactivation of the endocannabinoid system in obesity could be a deleterious effect, not only for metabolic but also for cardiovascular parameters.

The OZR model has been widely used to investigate the cardiovascular effects of obesity and insulin resistance. The cause of obesity in this model is a mutation of the fa gene, which determines the synthesis of leptin receptors. Homozygotes (fa/fa) with this mutation exhibit impaired responsiveness to leptin and become notably obese at 3 to 4 weeks of age [47]. It is important to consider that although the cause of obesity in OZRs is not common among humans, the phenotype parallels human obesity in many ways. These rats display increased triglycerides, cholesterol and insulin levels [47], are mildly hypertensive [48], [49] and eventually develop type 2 diabetes [47]. It must be noted, however, that at the age the OZRs were used in the current study, they did not display increased BP or blood glucose concentration. Therefore, the interference of these parameters in our results can be excluded**.**

One important finding in the current study was that although young OZRs displayed an overall impairment in vascular function, demonstrated by the reduced endothelium-dependent and endothelium-independent vasodilation (tested with ACh and SNP, respectively), specific mechanisms associated with cannabinoid signalling in the endothelium were also impaired in this model. This is supported by our findings showing that reduced anandamide-induced relaxation in mesenteric arteries from OZRs was only observed in endothelium-intact preparations. Considering that CB_1_ and CB_2_ receptors mediate anandamide-induced responses and the fact that protein expression of these receptors is decreased in both smooth muscle and endothelial cells of mesenteric arteries from OZRs (demonstrated by Western blotting and immunofluorescence analysis), we suggest that the reduction of anandamide response in OZRs might involve a decreased activation of CB_1_ and CB_2_ receptors by anandamide. In fact, endothelium-intact arteries from OZRs displayed decreased vascular relaxation to CB_1_ and CB_2_ agonists.

The mechanisms through which obesity directly causes vascular dysfunction are still an area of research. Human and animal studies have indicated the role of adipose tissue derived factors (including adipokines and cytokines), neurohumoral pathways, abnormalities in metabolic functions and modulation of pressor/depressor mechanisms, or even the combination or overlap of a number of these factors [50]. Based on the results from our study and considering previous studies demonstrating increased activation of the endocannabinoid system in obesity [8]–[10], we have now an important question: “How does increased adiposity in the absence of other pathologies affect vascular response to cannabinoid agonists?” Taking into consideration that increased visceral adiposity is considered as the ‘link’ between overweight and cardiometabolic complications and that a dysregulated, overstimulated endocannabinoid system is observed in visceral adipose tissue and appears to significantly contribute to the cardiometabolic complications of visceral obesity [51], [52], our novel data allow us to suggest that chronically overactive endocannabinoid system in visceral adipose tissue is related to vascular damage in OZRs. This leads to decreased vascular relaxation to cannabinoid receptor agonists as a consequence of down regulation of cannabinoid receptors and the related signaling pathways.

It is important to include in this discussion the fact that the effects of increased release of anandamide have been demonstrated only in tissues that control the metabolic homeostasis [8]–[10], where the protein expression of the CB1 receptor is increased, differently from that found in the present study. Considering this, the differential effects of obesity in the mesenteric vessels might constitute an important evidence for the impairment of this system in mesenteric arteries besides the increased release of anandamide in obesity. However, further studies are required to elucidate this.

Considering that the endothelium plays a role on the reduced relaxation to anandamide in mesenteric arteries from OZRs, a more detailed investigation of the NO-dependent relaxant pathway was carried out. Although the downstream signalling cascades regulated by cannabinoid receptor activation have been reported in neuronal cells, very little is known about the signalling cascades that are activated by cannabinoid agonists in the vasculature. There is evidence that anandamide-induced relaxation in resistance vessels is dependent on NO [21], [53]. In fact, our western blotting analysis demonstrated increased phosphorylation of eNOS in mesenteric arteries from LZRs after incubation with anandamide. In contrast, anandamide-induced phosphorylation of eNOS was decreased in arteries from OZRs. Therefore the involvement of a dysfunctional eNOS on the reduced response to anandamide in mesenteric arteries from OZRs is proposed in the present study.

Phosphorylation of eNOS plays a critical role in the regulation of NO production [54]. Multiple protein kinases, including AMPK [55], have been implicated in eNOS phosphorylation at Ser1177. Recent studies indicate that cannabinoid compounds activate the AMPK pathway in tissues that control energy homeostasis [56]. We here demonstrated activation of this protein by anandamide in mesenteric arteries from LZRs. Incubation of mesenteric arteries with anandamide resulted in a robust increase in phosphorylation of AMPK and ACC (the primary target of activated AMPK) in LZRs. The involvement of AMPK on the decreased relaxation response of mesenteric arteries to anandamide in obesity was also demonstrated in the present study. This is supported by the finding that activation of AMPK by AICAR corrected the decreased relaxation to anandamide in OZRs. The decreased anandamide-induced phosphorylation of both AMPK and ACC in mesenteric arteries from OZRs also indicates a potential dependence of AMPK on the decreased activation of eNOS in OZRs.

The link between AMPK and eNOS activation has been extensively studied. Kemp and colleagues first showed that AMPK phosphorylates eNOS at serine-1177, leading to activation of this enzyme [57]. The role of AMPK phosphorylation mediating eNOS activation by anandamide was confirmed by the observation that inhibition of AMPK with compound C abolished the anandamide-induced eNOS phosphorylation in mesenteric arteries from LZRs, indicating that anandamide-induced relaxation in LZRs depends, at least partially, on the activation of AMPK. In contrast, AMPK inhibition with compound C did not change the degree of eNOS phosphorylation in OZRs, which further supports our hypothesis that an impairment of AMPK activation is involved in the reduced anandamide-induced phosphorylation of eNOS in vessels from these animals. To date, no study has assessed the involvement of AMPK on the vascular responses to anandamide or the role of these pathways in alterations of cannabinoid responses in obesity.

Although the effects of AMPK activation was linked to NO production in the present study, it is important to mention the fact that anandamide-induced relaxation was barely reduced by NOS inhibition in LZRs and it was not affected in OZRs. There is clear evidence that the endothelium-derived relaxating factors work co-operatively in an integrated manner to maintain the health of the vasculature. In conditions where EDRFs production/bioavailability is impaired, a compensatory hypersensitivity of smooth muscle to relaxating factors occurs [58]. Considering this, it is possible that compensatory mechanisms are activated in LZRs when NOS is inhibited, contributing to the relatively small, overall reduction in anandamide relaxation in the presence of L-NAME.

The actions of cannabinoid compounds are terminated through intracellular enzymatic hydrolysis [28]. In particular; anandamide is rapidly metabolized by the FAAH to yield arachidonic acid and ethanolamine [11]. Since pretreatment of mesenteric arteries from OZRs with the selective FAAH inhibitor (URB597) corrected the deficit in anandamide-induced vascular relaxation, an increased degradation of anandamide in mesenteric arteries from OZRs might contribute to the decreased response to this agonist in obesity.

Besides interacting with its cognate receptors CB_1_ and CB_2_, anandamide can also activate vanilloid receptors on capsaicin-sensitive perivascular sensory nerves, and this interaction was shown to result in the release of the potent vasodilator peptide CGRP, evoking vasorelaxation [59]. Accordingly, in vitro treatment with capsaicin, which induces desensitization of C-fibers, profoundly suppressed anandamide responses of vessels from both LZRs and OZRs. Thus, it appears that effective silencing of vascular sensory C-fibers prevents blood vessels from responding normally to anandamide. Additionally, because the inhibition of anandamide responses of mesenteric arteries from OZRs was lower than that observed in samples from LZRs, we suggest that the impaired vascular response to anandamide in arteries from OZRs also involves decreased activation of C-fibre nerve endings. The functional implications of vascular C-fibre activation were previously demonstrated by Kawasaki in 1988 [60]. In addition, many other groups have demonstrated the vascular effects of C-fibre activation in different vascular preparations, including the rat mesenteric vascular bed [31]. However, the role of these nerves in the physiological regulation of vascular tone has remained uncertain. Recently, TRPV1 has emerged as a major site for activation of C-fibres in the periphery [61], and the presence of these receptors was previously demonstrated in nerves penetrating the walls of resistance mesenteric arteries. Although the protein expression of TRPV-1 was not altered in vessels from OZRs compared with samples from LZRs, we demonstrated the dependence of TRPV1 activation on anandamide-induced vessel relaxation using agents that interfere with vanilloid receptor activity, namely capsazepine [34] and ruthenium red [35]. This finding was confirmed using a CGRP receptor antagonist, which promoted a considerable decrease in anandamide responses of mesenteric arteriess from both LZRs and OZRs. However, the inhibition observed with all of these blockers was lower in OZRs. These findings, coupled with the observation of the decreased relaxation to capsaicin in OZRs, indicate that impairment of the anandamide responses mediated by vanilloid receptors activation is also present in OZRs and might contribute to the vascular dysfunction present in this model.

In conclusion, the present study demonstrates that relaxation induced by the cannabinoid agonist anandamide is decreased in endothelium-intact arteries from young OZRs. Reduced cannabinoid receptors expression, decreased anandamide-induced activation of AMPK and eNOS, increased degradation of anandamide as well as impairment of the response mediated by TRPV1 activation might be involved in the decreased response to anandamide in OZRs.

Our findings further underscore the relationship between obesity and vascular dysfunction and may provide new evidence for a role of the endocannabinoid system as one of the mechanisms accounting for the overall impairment of the vascular function in obesity.

## References

[1] Flegal KM, Carroll MD, Ogden CL, Johnson CL (2002) Prevalence and trends in obesity among US adults, 1999–2000. JAMA 288 (14): 1723–1727. 10.1001/jama.288.14.1723 12365955

[2] Hill JO (2006) Understanding and addressing the epidemic of obesity: an energy balance perspective. Endocr Rev 27 (7): 750–761. 10.1210/er.2006-0032 17122359

[3] Echahidi N, Mohty D, Pibarot P, Despres JP, O’Hara G, et al.. (2007) Obesity and metabolic syndrome are independent risk factors for atrial fibrillation after coronary artery bypass graft surgery. Circulation 116 (11 Suppl): I213–219. 10.1161/CIRCULATIONAHA.106.681304 17846306

[4] Stapleton PA, James ME, Goodwill AG, Frisbee JC (2008) Obesity and vascular dysfunction. Pathophysiology 15 (2): 79–89. 10.1016/j.pathophys.2008.04.007 PMC2593649 18571908

[5] Gokce N, Keaney JF, Hunter LM, Watkins MT, Nedeljkovic ZS, et al (2003) Predictive value of noninvasively determined endothelial dysfunction for long-term cardiovascular events in patients with peripheral vascular disease. J Am Coll Cardiol 41 (10): 1769–1775. 10.1016/s0735-1097(03)00333-4 12767663

[6] Lerman A, Zeiher AM (2005) Endothelial function: cardiac events. Circulation 111 (3): 363–368. 10.1161/01.CIR.0000153339.27064.14 15668353

[7] Mitchell GF, Parise H, Vita JA, Larson MG, Warne E, et al (2004) Local shear stress and brachial artery flow-mediated dilation: the Framingham Heart Study. Hypertension 44 (2): 134–139. 10.1161/01.HYP.0000137305.77635.68 15249547

[8] Engeli S, Bohnke J, Feldpausch M, Gorzelniak K, Janke J, et al (2005) Activation of the peripheral endocannabinoid system in human obesity. Diabetes 54 (10): 2838–2843. 10.2337/diabetes.54.10.2838 PMC2228268 16186383

[9] Matias I, Petrosino S, Racioppi A, Capasso R, Izzo AA, et al (2008) Dysregulation of peripheral endocannabinoid levels in hyperglycemia and obesity: Effect of high fat diets. Mol Cell Endocrinol 286 (1–2 Suppl 1)S66–78. 10.1016/j.mce.2008.01.026 18343566

[10] Szmitko PE, Verma S (2008) The endocannabinoid system and cardiometabolic risk. Atherosclerosis 199 (2): 248–256. 10.1016/j.atherosclerosis.2008.03.011 18440538

[11] Cravatt BF, Giang DK, Mayfield SP, Boger DL, Lerner RA, et al (1996) Molecular characterization of an enzyme that degrades neuromodulatory fatty-acid amides. Nature 384 (6604): 83–87. 10.1038/384083a0 8900284

[12] Di Marzo V (2008) Targeting the endocannabinoid system: to enhance or reduce? Nat Rev Drug Discov 7 (5): 438–455. 10.1038/nrd2553 18446159

[13] Liu J, Wang L, Harvey-White J, Huang BX, Kim HY, et al (2008) Multiple pathways involved in the biosynthesis of anandamide. Neuropharmacology 54 (1): 1–7. 10.1016/j.neuropharm.2007.05.020 PMC2219543 17631919

[14] Mackie K, Stella N (2006) Cannabinoid receptors and endocannabinoids: evidence for new players. AAPS J 8 (2): E298–306. 10.1007/BF02854900 PMC3231556 16796380

[15] Cota D, Marsicano G, Tschop M, Grubler Y, Flachskamm C, et al (2003) The endogenous cannabinoid system affects energy balance via central orexigenic drive and peripheral lipogenesis. J Clin Invest 112 (3): 423–431. 10.1172/JCI17725 PMC166293 12897210

[16] Gardiner SM, March JE, Kemp PA, Bennett T (2009) Factors influencing the regional haemodynamic responses to methanandamide and anandamide in conscious rats. Br J Pharmacol 158 (4): 1143–1152. 10.1111/j.1476-5381.2009.00363.x PMC2785535 19702785

[17] Wheal AJ, Bennett T, Randall MD, Gardiner SM (2007) Cardiovascular effects of cannabinoids in conscious spontaneously hypertensive rats. Br J Pharmacol 152 (5): 717–724. 10.1038/sj.bjp.0707410 PMC2190006 17700721

[18] Grainger J, Boachie-Ansah G (2001) Anandamide-induced relaxation of sheep coronary arteries: the role of the vascular endothelium, arachidonic acid metabolites and potassium channels. Br J Pharmacol 134 (5): 1003–1012. 10.1038/sj.bjp.0704340 PMC1573033 11682448

[19] Herradon E, Martin MI, Lopez-Miranda V (2007) Characterization of the vasorelaxant mechanisms of the endocannabinoid anandamide in rat aorta. Br J Pharmacol 152 (5): 699–708. 10.1038/sj.bjp.0707404 PMC2190007 17704831

[20] Lipez-Miranda V, Herradon E, Martin MI (2008) Vasorelaxation caused by cannabinoids: mechanisms in different vascular beds. Curr Vasc Pharmacol 6 (4): 335–346. 10.2174/157016108785909706 18855721

[21] Deutsch DG, Goligorsky MS, Schmid PC, Krebsbach RJ, Schmid HH, et al (1997) Production and physiological actions of anandamide in the vasculature of the rat kidney. J Clin Invest 100 (6): 1538–1546. 10.1172/JCI119677 PMC508335 9294122

[22] Wagner JA, Varga K, Járai Z, Kunos G (1999) Mesenteric vasodilation mediated by endothelial anandamide receptors. Hypertension 33 (1 Pt 2): 429–34. 10.1161/01.hyp.33.1.429 9931142

[23] Hillard CJ (2000) Endocannabinoids and vascular function. J Pharmacol Exp Ther 294 (1): 27–32. 10871291

[24] Randall MD, Kendall DA, O’Sullivan S (2004) The complexities of the cardiovascular actions of cannabinoids. Br J Pharmacol 142 (1): 20–26. 10.1038/sj.bjp.0705725 PMC1574918 15131000

[25] Pratt PF, Hillard CJ, Edgemond WS, Campbell WB (1998) N-arachidonylethanolamide relaxation of bovine coronary artery is not mediated by CB1 cannabinoid receptor. Am J Physiol 274 (1 Pt 2): H375–81. 10.1152/ajpheart.1998.274.1.H375 9458889

[26] Mulvany MJ, Halpern W (1977) Contractile properties of small arterial resistance vessels in spontaneously hypertensive and normotensive rats. Circ Res 41 (1): 19–26. 10.1161/01.res.41.1.19 862138

[27] O’Sullivan SE, Kendall DA, Randall MD (2004) Heterogeneity in the mechanisms of vasorelaxation to anandamide in resistance and conduit rat mesenteric arteries. Br J Pharmacol 142 (3): 435–442. 10.1038/sj.bjp.0705810 PMC1574972 15148250

[28] Jiang S, Fu Y, Williams J, Wood J, Pandarinathan L, et al (2007) Expression and function of cannabinoid receptors CB1 and CB2 and their cognate cannabinoid ligands in murine embryonic stem cells. PLoS One 2 (7): e641. 10.1371/journal.pone.0000641 PMC1919431 17653268

[29] Kaczocha M, Glaser ST, Deutsch DG (2009) Identification of intracellular carriers for the endocannabinoid anandamide. Proc Natl Acad Sci USA 106 (15): 6375–6380. 10.1073/pnas.0901515106 PMC2669397 19307565

[30] Goirand F, Solar M, Athea Y, Viollet B, Mateo P, et al (2007) Activation of AMP kinase alpha1 subunit induces aortic vasorelaxation in mice. J Physiol 581 (Pt 3): 1163–1171. 10.1113/jphysiol.2007.132589 PMC2170850 17446219

[31] Lobato NS, Filgueira FP, Akamine EH, Davel AP, Rossoni LV, et al (2011) Obesity induced by neonatal treatment with monosodium glutamate impairs microvascular reactivity in adult rats: role of NO and prostanoids. Nutr Metab Cardiovasc Dis 21 (10): 808–16. 10.1016/j.numecd.2010.02.006 20554176

[32] Holzer P (1991) Capsaicin: cellular targets, mechanisms of action, and selectivity for thin sensory neurons. Pharmacol Rev 43 (2): 143–201. 1852779

[33] Scotland RS, Chauhan S, Davis C, De Felipe C, Hunt S, et al (2004) Vanilloid receptor TRPV1, sensory C-fibers, and vascular autoregulation: a novel mechanism involved in myogenic constriction. Circ Res 95 (10): 1027–1034. 10.1161/01.RES.0000148633.93110.24 15499026

[34] Caterina MJ, Schumacher MA, Tominaga M, Rosen TA, Levine JD, et al (1997) The capsaicin receptor: a heat-activated ion channel in the pain pathway. Nature 389 (6653): 816–824. 10.1038/39807 9349813

[35] Bevan S, Hothi S, Hughes G, James IF, Rang HP, et al (1992) Capsazepine: a competitive antagonist of the sensory neurone excitant capsaicin. Br J Pharmacol 107 (2): 544–552. 10.1111/j.1476-5381.1992.tb12781.x PMC1907893 1422598

[36] Dray A, Forbes CA, Burgess GM (1990) Ruthenium red blocks the capsaicin-induced increase in intracellular calcium and activation of membrane currents in sensory neurones as well as the activation of peripheral nociceptors in vitro. Neurosci Lett 110 (1–2): 52–59. 10.1016/0304-3940(90)90786-9 1691472

[37] Zygmunt PM, Andersson DA, Hogestatt ED (2002) Delta 9-tetrahydrocannabinol and cannabinol activate capsaicin-sensitive sensory nerves via a CB1 and CB2 cannabinoid receptor-independent mechanism. J Neurosci 22 (11): 4720–4727. 10.1523/JNEUROSCI.22-11-04720.2002 PMC6758782 12040079

[38] Rayment SJ, Ralevic V, Barrett DA, Cordell R, Alexander SP (2007) A novel mechanism of vasoregulation: ADP-induced relaxation of the porcine isolated coronary artery is mediated via adenosine release. FASEB J 21 (2): 577–585. 10.1096/fj.06-7050com 17167068

[39] Ralevic V, Kendall DA, Randall MD, Zygmunt PM, Movahed P, et al (2000) Vanilloid receptors on capsaicin-sensitive sensory nerves mediate relaxation to methanandamide in the rat isolated mesenteric arterial bed and small mesenteric arteries. Br J Pharmacol 130: 1483–1488. 10928948 10.1038/sj.bjp.0703456 PMC1572215

[40] Wagner JA, Varga K, Ellis EF, Rzigalinski BA, Martin BR, et al (1997) Activation of peripheral CB1 cannabinoid receptors in haemorrhagic shock. Nature 390(6659): 518–21. 9394002 10.1038/37371

[41] Varga K, Wagner JA, Bridgen DT, Kunos G (1998) Platelet- and macrophage-derived endogenous cannabinoids are involved in endotoxin-induced hypotension. FASEB J. 12(11): 1035–44. 10.1096/fasebj.12.11.1035 9707176

[42] Kadoi Y, Hinohara H, Kunimoto F, Saito S, Goto F (2005) Cannabinoid antagonist AM 281 reduces mortality rate and neurologic dysfunction after cecal ligation and puncture in rats. Crit Care Med. 33(11): 2629–2636. 10.1097/01.ccm.0000187010.14426.cc 16276190

[43] Wagner JA, Hu K, Bauersachs J, Karcher J, Wiesler M, et al (2001) Endogenous cannabinoids mediate hypotension after experimental myocardial infarction. J Am Coll Cardiol. 38(7): 2048–2054. 10.1016/s0735-1097(01)01671-0 11738314

[44] Gaskari SA, Liu H, Moezi L, Li Y, Baik SK, et al (2005) Role of endocannabinoids in the pathogenesis of cirrhotic cardiomyopathy in bile duct-ligated rats. Br J Pharmacol. 146(3): 315–23. 10.1038/sj.bjp.0706331 PMC1576281 16025138

[45] Mukhopadhyay P, Bátkai S, Rajesh M, Czifra N, Harvey-White J, et al (2007) Pharmacological inhibition of CB1 cannabinoid receptor protects against doxorubicin-induced cardiotoxicity. J Am Coll Cardiol. 50(6): 528–536. 10.1016/j.jacc.2007.03.057 PMC2239316 17678736

[46] Bátkai S, Pacher P, Osei-Hyiaman D, Radaeva S, Liu J, et al (2004) Endocannabinoids acting at cannabinoid-1 receptors regulate cardiovascular function in hypertension. Circulation. 110(14): 1996–2002. 10.1161/01.CIR.0000143230.23252.D2 PMC2756479 15451779

[47] Sarzani R (2008) Endocannabinoids, blood pressure and the human heart. J Neuroendocrinol. 20(1): 58–62. 10.1111/j.1365-2826.2008.01677.x 18426501

[48] Zucker LM, Antoniades HN (1972) Insulin and obesity in the Zucker genetically obese rat “fatty”. Endocrinology 90 (5): 1320–1330. 10.1210/endo-90-5-1320 5012744

[49] Stepp DW, Boesen EI, Sullivan JC, Mintz JD, Hair CD, et al (2007) Obesity augments vasoconstrictor reactivity to angiotensin II in the renal circulation of the Zucker rat. Am J Physiol Heart Circ Physiol 293 (4): H2537–H2542. 10.1152/ajpheart.01081.2006 17693541

[50] Subramanian R, MacLeod KM (2003) Age-dependent changes in blood pressure and arterial reactivity in obese Zucker rats. Eur J Pharmacol 477 (2): 143–152. 10.1016/j.ejphar.2003.08.003 14519418

[51] Kotsis V, Stabouli S, Papakatsika S, Rizos Z, Parati G (2010) Mechanisms of obesity-induced hypertension. Hypertens Res. 33(5): 386–393. 10.1038/hr.2010.9 20442753

[52] Blüher M, Engeli S, Klöting N, Berndt J, Fasshauer M, et al (2006) Dysregulation of the peripheral and adipose tissue endocannabinoid system in human abdominal obesity. Diabetes. 55(11): 3053–60. 10.2337/db06-0812 PMC2228260 17065342

[53] Côté M, Matias I, Lemieux I, Petrosino S, Alméras N, et al (2007) Circulating endocannabinoid levels, abdominal adiposity and related cardiometabolic risk factors in obese men. Int J Obes 31(4): 692–699. 10.1038/sj.ijo.0803539 17224929

[54] Randall MD, Kendall DA, O’Sullivan S (2004) The complexities of the cardiovascular actions of cannabinoids. Br J Pharmacol 142 (1): 20–26. 10.1038/sj.bjp.0705725 PMC1574918 15131000

[55] Shaul PW (2002) Regulation of endothelial nitric oxide synthase: location, location, location. Annu Rev Physiol 64: 749–774. 11826287 10.1146/annurev.physiol.64.081501.155952

[56] Chen Z, Peng IC, Sun W, Su MI, Hsu PH, et al (2009) AMP-activated protein kinase functionally phosphorylates endothelial nitric oxide synthase Ser633. Circ Res 104 (4): 496–505. 10.1161/CIRCRESAHA.108.187567 PMC2761102 19131647

[57] Kola B, Hubina E, Tucci SA, Kirkham TC, Garcia EA, et al (2005) Cannabinoids and ghrelin have both central and peripheral metabolic and cardiac effects via AMP-activated protein kinase. J Biol Chem 280 (26): 25196–25201. 10.1074/jbc.C500175200 15899896

[58] Chen ZP, Mitchelhill KI, Michell BJ, Stapleton D, Rodriguez-Crespo I, et al (1999) AMP-activated protein kinase phosphorylation of endothelial NO synthase. FEBS Lett 443 (3): 285–289. 10.1016/s0014-5793(98)01705-0 10025949

[59] Moncada S, Rees DD, Schulz R, Palmer RM (1991) Development and mechanism of a specific supersensitivity to nitrovasodilators after inhibition of vascular nitric oxide synthesis in vivo. Proc Natl Acad Sci U S A 88 (6): 2166–2170. 10.1073/pnas.88.6.2166 PMC51190 1848694

[60] Zygmunt PM, Petersson J, Andersson DA, Chuang H, Sorgard M, et al (1999) Vanilloid receptors on sensory nerves mediate the vasodilator action of anandamide. Nature 400 (6743): 452–457. 10.1038/22761 10440374

[61] Kawasaki H, Takasaki K, Saito A, Goto K (1988) Calcitonin gene-related peptide acts as a novel vasodilator neurotransmitter in mesenteric resistance vessels of the rat. Nature 335 (6186): 164–167. 10.1038/335164a0 2901042

[62] Gunthorpe MJ, Benham CD, Randall A, Davis JB (2002) The diversity in the vanilloid (TRPV) receptor family of ion channels. Trends Pharmacol Sci 23 (4): 183–191. 10.1016/s0165-6147(02)01999-5 11931994

